# Warming has stronger direct than indirect effects on benthic microalgae in a seaweed system in spring

**DOI:** 10.1007/s00227-017-3109-x

**Published:** 2017-03-06

**Authors:** Franziska Julie Werner, Birte Matthiessen

**Affiliations:** GEOMAR Helmholtz Centre for Ocean Research Kiel, Experimental Ecology and Food Webs, Düsternbrooker Weg 20, 24105 Kiel, Germany

## Abstract

Using outdoor mesocosms we investigated the relative importance of the direct and indirect (here: altered grazing) effects of seawater warming on benthic microalgae in a Baltic Sea *Fucus vesiculosus* (Phaeophyceae) system during the spring season. Seawater warming had a positive main effect on microalgal total biomass accrual and growth rate and on total mesograzer abundance and biomass. Moreover, under the existing resource-replete conditions in spring the direct positive effect of warming on microalgae was stronger than its indirect negative effect through enhanced grazing. The outcome of this study contrasts previous observations from the summer and winter season, where indirect effects of warming mediated by altered grazing were identified as an important driver of primary biomass in the *Fucus* system. In this context, the results from the spring season add mechanistic information to the overall understanding of the seasonal variability of climate change effects. They suggest that the relative importance of the underlying direct and indirect effective pathways of warming and the overall effect on the balance between production and consumption are influenced by the trophic state of the system, which in temperate regions is related to season.

**Electronic supplementary material:**

The online version of this article (doi:10.1007/s00227-017-3109-x) contains supplementary material, which is available to authorized users.

## Introduction

Climate change research on marine systems has increasingly provided evidence that indirect effects mediated by altered species interactions can play a key role in driving an ecosystem’s overall response to global climate change (e.g., Schiel et al. 2004; Traill et al. 2010; Kordas et al. 2011; Alsterberg et al. 2013; Falkenberg et al. 2014). Particularly, top-down control (grazing) was identified as a crucial interface where direct effects of rising seawater temperature translate into indirect effects on primary producers with regard to algal size fractionation and community composition (Sommer and Lengfellner 2008; Sommer and Lewandowska 2011) and overall biomass production (Sommer and Lengfellner 2008; Alsterberg et al. 2013; Falkenberg et al. 2014; Werner et al. 2016a). This indirect pathway of temperature effects was explained by the metabolic theory of ecology (MTE) stating that (bio)chemical reactions, in general, are stimulated by temperature with metabolic processes of heterotrophs such as feeding, growth and reproduction being activated more strongly than photosynthetic rates of autotrophs (Brown et al. 2004; Allen et al. 2005; Lopez-Urrutia et al. 2006; O’Connor 2009; Carr and Bruno 2013). Based on this, it is generally assumed that marine food webs may face a shift in balance between autotrophic production and heterotrophic consumption under global warming with potential consequences for the structure and functioning of the associated ecosystem. However, studies involving multiple species across trophic levels that clearly test the relative importance of both direct and indirect temperature effects on primary biomass are still scarce.

We set out to experimentally disentangle the direct and indirect effects of elevated seawater temperature on benthic microalgae in a Baltic Sea seaweed (*Fucus vesiculosus*, Phaeophyceae) system. Generally, benthic microalgae that grow on hard substrate (epilithic) or on the surface of macrophytes (epiphytic) exert an important structuring control over perennial seaweed and seagrass beds of coastal marine habitats. Together with ephemeral filamentous macroalgae, they function as an important food source at the base of the macrophyte-associated food web (Miller et al. 1996; Underwood and Kromkamp 1999; Lebreton et al. 2011), but then can also impede functioning of the system through outcompeting (for nutrients and light) and ultimately overgrowth of the foundation macrophyte (Sand-Jensen 1977; Wallentinus 1984; Schramm and Nienhuis 1996; Worm et al. 1999). Dominance of competitively superior fast-growing microalgae in macrophyte systems is counterbalanced by the top-down control of mesograzers (e.g., Worm et al. 1999, 2000; Burkepile and Hay 2006; Valentine and Duffy 2006). In *F. vesiculosus* stands of the southwestern Baltic Sea, the gastropod *Littorina littorea* and the crustaceans *Idotea* spp. and *Gammarus* spp. constitute the most abundant mesograzers with complementary feeding modes and preferences (Lotze 1998; Sommer 1999a, b; Worm et al. 1999). With regard to microalgal biofilms, *L. littorea* exerts the most efficient grazing control by leaving algal-cleared feeding tracks on the substrate (Steneck and Watling 1982; Sommer 1999a, 2000).

Recent experimental work showed that the ecological balance between competition (bottom-up control) and consumption (top-down control) in the *F. vesiculosus* system can be disrupted by elevated seawater temperature (Werner et al. 2016a, b). Precisely, it was shown that temperature-induced alteration of top-down grazing functions as a key driver of primary producer biomass under global change scenarios (Werner et al. 2016a, b). Warming (Δ +5 °C), however, did not generally strengthen top-down control as is commonly assumed based on MTE. Whereas, prolonged warmer temperatures in winter intensified consumption, warming in summer exceeded the thermal tolerance limit of two (*Gammarus* spp. and *Idotea* spp.) of the three predominant mesograzer taxa, leading to significantly weakened top-down control and to intensified overgrowth of the foundation seaweed *F. vesiculosus* by epiphytic microalgae and filamentous macroalgae. While the temperature effects on algal biomass seemed considerably indirectly driven by altered top-down grazing, the direct and indirect pathways could not be quantitatively partitioned at this point. According to MTE both heterotrophic metabolism and photosynthesis are stimulated by temperature, although at different activation rates (Brown et al. 2004; Allen et al. 2005; Rall et al. 2010). Under sufficient resource availability (e.g., inorganic nutrients and light) it is therefore possible that both the release from grazing pressure and the temperature-enhanced growth of competitively superior micro- and filamentous macroalgae caused the outcompeting of the seaweed.

To disentangle the direct and indirect effects of warming we conducted a study in spring 2015 using the same experimental set-up of the *F. vesiculosus* system (Werner et al. 2016a, b), while manipulating temperature and grazer presence in a factorial design. We hypothesized (1) that warming has a positive main effect on microalgal biomass accumulation and growth rate under the given resource-replete conditions in spring. Moreover, we expected this positive effect on algal biomass to be stronger in the absence of grazers, revealing the direct effect of temperature. Based on MTE predictions, we assumed (2) that seawater warming accelerates metabolic processes such as feeding, growth, and reproduction in the heterotrophic mesograzers and that this effect is reflected in increased total grazer abundance and total grazer biomass. In the light of the above, we expected (3) that mesograzers generally reduce the biomass of microalgae and that warming indirectly causes a stronger decrease of algal biomass as it intensifies grazing pressure. Lastly, on the base of our previous experimental findings, we expected (4) that warming affects the mesograzer taxa differently leading to taxon-specific responses in their abundance and biomass.

## Methods

### Experimental set-up

The experiment was conducted in the Kiel Outdoor Benthocosms from March 5th to April 15th 2015. The benthocosms comprise 12 experimental tanks (1.4 m^3^ each) that are located outdoors on a jetty in the Kiel Fjord, Germany. They are exposed to ambient light and weather conditions year-round. In this experiment all tanks were filled with non-filtered seawater taken from the Kiel Fjord, from 1 m depth in close vicinity to the experimental platform. The water body was replaced once per day via a flow-through system, which kept the ambient experimental conditions close to the environmental conditions of the Kiel Fjord. Temperature was regulated by heat exchangers and internal heating elements (Titan 2000, Aqua Medic, Bissendorf, Germany and Schego Titan, 600 W, Schemel and Goetz, Offenback/ Main, Germany) and was continuously logged (Profilux sensors 3ex, GHL Advanced Technology, Kaiserslautern, Germany). A more detailed technical description of the Kiel Outdoor Benthocosms, their installation, programming and monitoring can be found in Wahl et al. (2015). To keep this study comparable to former experiments, *F. vesiculosus* communities (i.e., 20 thalli of the seaweed on its natural rock substratum, associated micro- and filamentous macroalgae, and mesograzers) were established in the benthocosms according to Werner et al. (2016a). Additionally, for the quantitative sampling of microalgae, one PVC plate (0.60 × 0.40 m) holding unglazed ceramic tiles (4.5 × 4.5 cm) was hung in the *F. vesiculosus* stands in 20 cm depth in each experimental tank (see below). All tiles were facing the same direction and had been pre-colonized by microalgae in the Kiel Fjord for 10 days. Similar microalgal starting biomass on the tiles was ensured by testing a subsample of three randomly selected tiles for their chlorophyll *a* content (for method see below) prior to the distribution of the tiles into each experimental unit (tank).

### Treatments

**Fig. 1:**
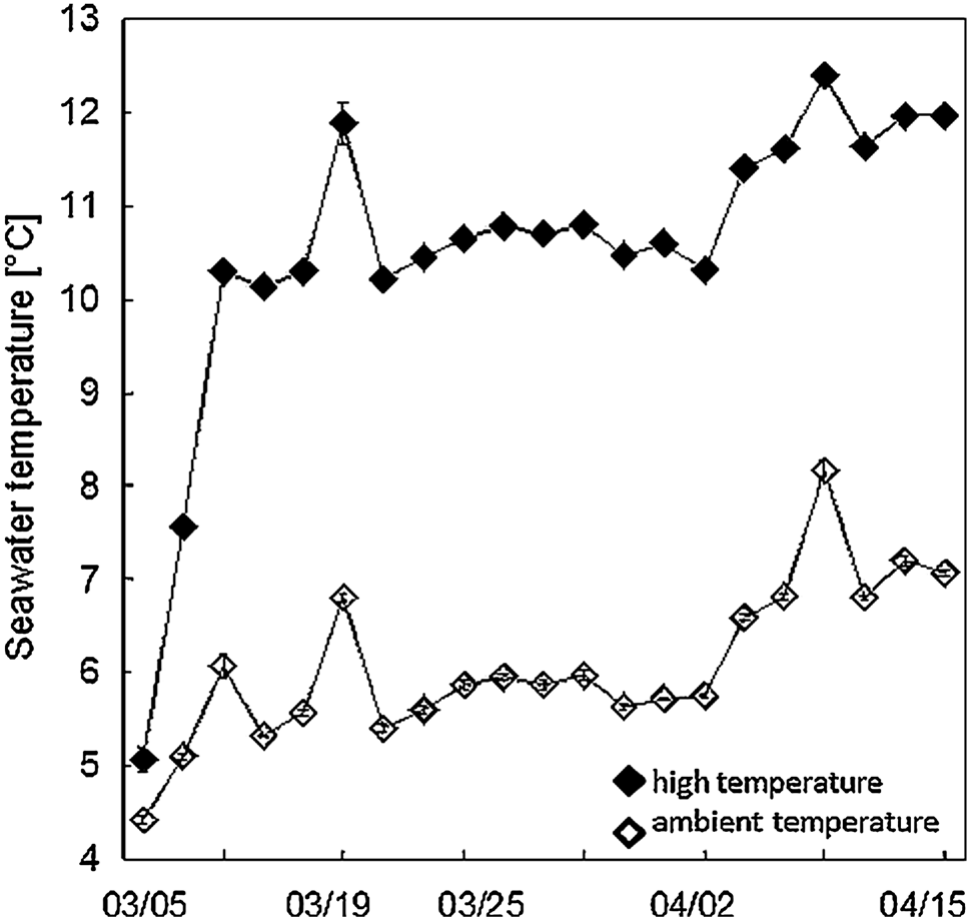
Display of the seawater temperature (°C) in ambient (*open diamonds*) and high (*filled diamonds*) temperature treatments over the course of the experimental runtime from March 5th to April 15th 2015.

In the experiment two levels of temperature (ambient vs. elevated) and grazers (present vs. absent) were full-factorially crossed. The temperature manipulation comprised +5 °C according to climate change predictions for the Baltic Sea region (HELCOM 2007; BACC 2008, 2015; Schernewski et al. 2010). The ambient and elevated temperature treatments did not describe a fixed value, but were allowed to follow diurnal and seasonal fluctuation while Δ*T* = +5 °C was maintained (Fig. 1; Wahl et al. 2015). For the grazer manipulation, the three most important mesograzers of the *F. vesiculosus* system (*Idotea* spp., *Gammarus* spp., *Littorina littorea*) were collected, sorted into approximate size classes, and counted. 29 individuals of *Idotea* spp., 225 *Gammarus* spp., and 48 *L. littorea* were added to each one of half of the experimental tanks. The initial number of mesograzers reflected their natural abundance found in the field during the spring season (compare Werner et al. 2016a).

The experimental design resulted in a total of four treatment combinations that were replicated three times. The no-grazer treatment (−G) reflected the ambient seawater temperature conditions of the Kiel Fjord, while mesograzers were excluded from the experimental communities. The grazer treatment (+G) reflected the ambient temperature of the Kiel Fjord with grazers being present. The no-grazer and elevated temperature treatment (+T−G) comprised a delta value of Δ*T* = +5 °C relative to the ambient temperature treatment (see Fig. 1) in the absence of grazers. The grazer and elevated temperature treatment (+T+G) comprised the same temperature treatment in the presence of grazers.

### Sampling and response variables

Sampling took place after 6 weeks at the end of the experiment. During sampling all mesograzers were removed from the experimental tanks. They were identified, sorted, and counted. A random subsample of 15–20 individuals per grazer taxon and experimental unit was taken for the analysis of the total grazer biomass (mg AFDW without shell), and the per capita biomass of each grazer taxon (mg AFDW without shell individual^−1^).

Due to the patchy growth of microalgae on the surface of *F. vesiculosus*, the microalgal biomass response per treatment was analyzed based on the algae growing on the ceramic tiles (hanging in the seaweed stand of each tank). Microalgal total biomass was expressed as total chlorophyll *a* content (*µ*g cm^−2^) (hereafter Chl *a*). Microalgal growth was calculated as growth rate day^−1^ using Chl *a* measurements: $\mu ={\frac{\ln \left( {{N}_{2}} \right)}{\ln \left( {{N}_{1}} \right)}}/{({{t}_{2}}-{{t}_{1}})}\;$, where *N* and *t* are the microalgal biomass (Chl *a*) at times 2 and 1, respectively. For the analysis three randomly chosen tiles per tank were sampled. The microalgal material was scraped and rinsed off with a razor blade and a defined volume of sterile filtered seawater (75–80 mL, 0.2 µm), respectively. The removed algal material was pooled and homogenized per experimental unit. About 2 mL of the diluted sample was filtered on pre-combusted Whatman GF/F filters and stored at −20 °C until further analysis. Chl *a* analysis was conducted spectrophotometrically according to Jeffrey and Humphrey (1975). We are aware that the community composition of the microalgae on the seaweed surface and on the artificial substratum may slightly differ due to inhibiting mechanisms of the seaweed. Nonetheless, we observed that the most important genera contributing to total benthic microalgal biomass (e.g., *Achnanthes* spp., *Licmophora* spp., *Melosira* spp., *Navicula* spp., *Stauroneis* spp., *Synedra* spp.) largely overlap and that there is no significant difference in the microalgal community diversity on the surface of *F. vesiculosus* and ceramic tiles (see electronic supplementary material). Moreover, microalgae on both substrates share a common propagule pool and are subjected to the same structuring forces (bottom-up and top-down regulation) in the experimental system.

### Statistical analyses

Prior to the analysis, data were tested for normal distribution and homogeneity of variances. Data transformation was only necessary for the per capita biomass of the amphipod *Gammarus* spp. (log). A full factorial analysis of variance (ANOVA) was applied to test the main effect of temperature (T) and grazers (G) and their interaction on the total biomass accumulation and growth rate of the microalgae. To estimate the relative importance of each of the contributing factors, effect sizes were calculated as omega squared: ${{\omega }^{2}}=\text{S}{{\text{S}}_{\text{treatment}}}-d{{f}_{\text{treatment}}}\times {{M}_{\text{Serror}}}/\text{S}{{\text{S}}_{\text{total}}}+{{M}_{\text{Serror}}}$ (Olejnik and Algina 2003). A *t* test was then performed to analyze the effect of temperature on total grazer abundance, total grazer biomass, and the abundance and per capita biomass of each individual grazer taxon (i.e., *Gammarus* spp., *Idotea* spp., *L. littorea*).

To disentangle the direct and indirect (mediated by altered grazing) effects of temperature on algal biomass accumulation and growth rate, a priori planned comparisons of the treatments were conducted. More specifically, to test the direct effect of temperature on microalgal total biomass and growth rate according to hypothesis: (1), the respective response variables were compared in ambient against high temperature treatments under non-grazed (i.e., −G vs. +T−G) or grazed (i.e., +G vs. +T+G) conditions. Furthermore, to test the indirect effect of temperature (via altered grazing) on microalgal total biomass according to hypothesis (3), the response variable was compared in grazed against non-grazed treatments under either ambient or high temperature conditions (i.e., −G vs. +G and +T−G vs. +T+G). In order to account for alpha-inflation in the repeated comparison, Bonferroni correction was applied. This procedure rendered all results of the a priori comparison non-significant, which is a common problem in ecological studies as they often feature a small number of replicates and high within-sample variability (Moran 2003). In order to account for a potential type II error, we report both the outcome of the a priori comparison prior to correction and the adjusted critical *p*-level (Table 3) and carefully discuss and interpret the results. All statistical analyses were conducted using Statistica 6.1 (StatSoft Inc., Tulsa, Oklahoma, USA). The data set for this publication is available at https://doi.pangaea.de/10.1594/PANGAEA.872416.

## Results

**Fig. 2:**
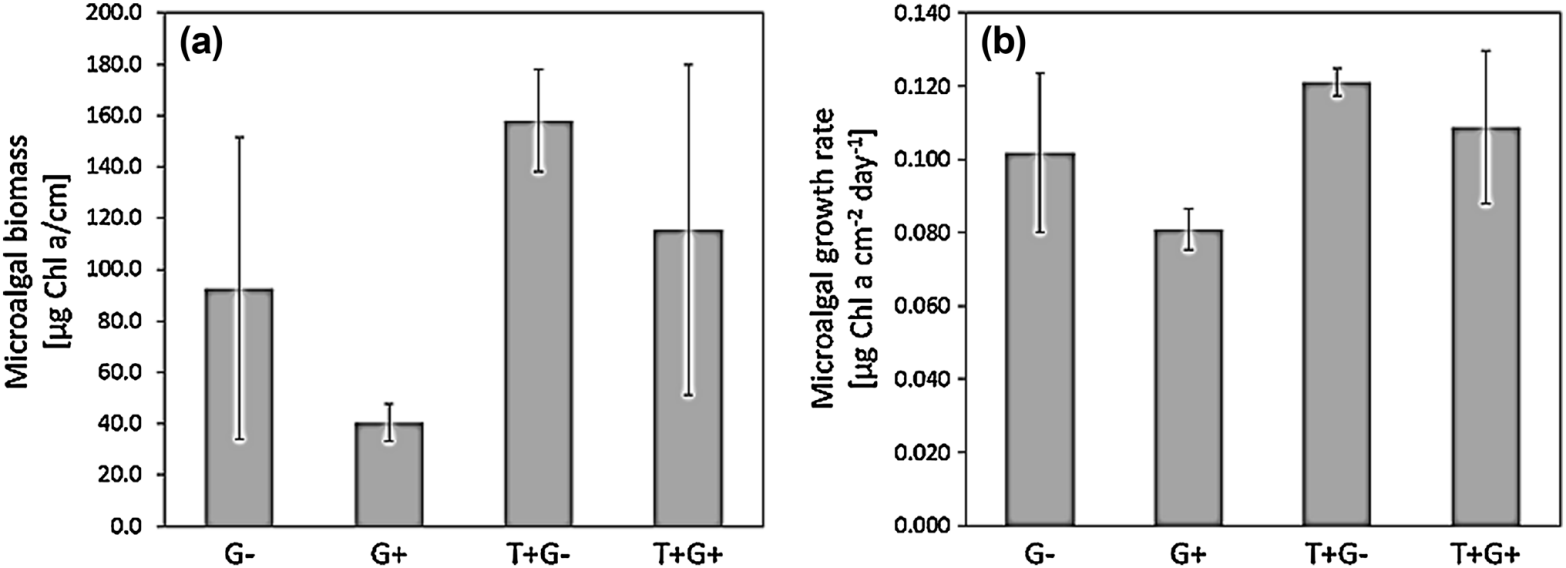
Display (mean ± CI) of microalgal biomass (**a**) and growth rate day^−1^ (**b**) measured as Chl *a* (µg cm^−2^). Responses are shown for all seawater temperature and grazer manipulations. Treatment combinations are shown as −G: ambient temperature/ grazer absent; +G: ambient temperature/ grazer present; +T−G: high temperature/ grazer absent; +T+G: high temperature/grazer present. The sample size (*N*) was twelve

**Fig. 3:**
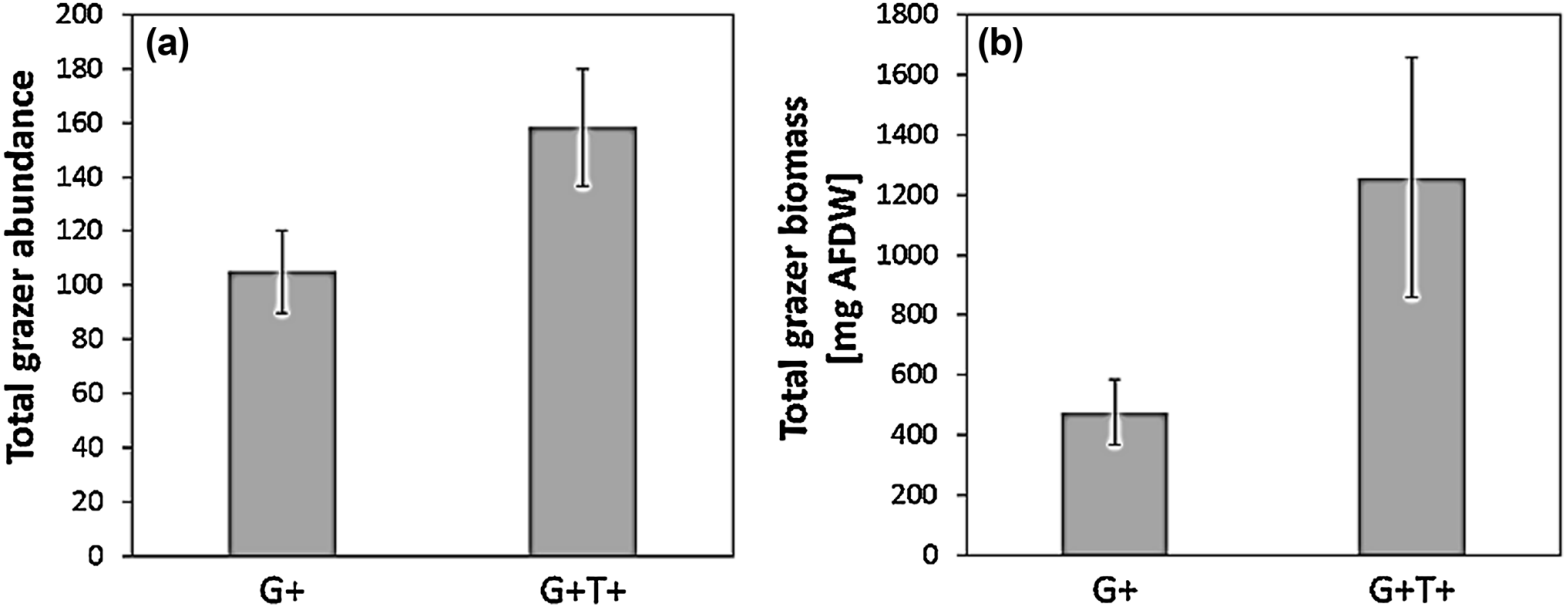
Display (mean ± CI) of the total grazer abundance (**a**) and the total grazer biomass (mg AFDW without shell) (**b**) in ambient (+G) and high temperature (+G+T) treatments. Sample size (*N*) was six

Elevated seawater temperature had a significant positive main effect on microalgal total biomass accrual and growth rate (Fig. 2; Table 1). Likewise, elevated seawater temperature had a significant positive effect on mesograzer total abundance and biomass (Fig. 3; Table 2). This positive effect of warming on consumers, however, did not translate into a significant negative effect on microalgae. Regardless of temperature, the presence of mesograzers only showed a trend of a negative main effect on microalgal total biomass and growth rate (Fig. 2; Table 1).

**Table 1:** ANOVA results explaining the effects of temperature (temp) and grazers and their interaction on microalgal total biomass (µg cm^−2^ Chl *a*) and on microalgal growth rate (µg cm^−2^ Chl *a* day^−1^). The effect size of each contributing factor is shown as omega squared (*Ѡ* ^2^). Sample size (*N*) was twelve

Variable	Factor	*df*	MS	*F*	*P*	*Ѡ* ^*2*^
Microalgae	Temp	1	924.05	9.419	0.015	0.37
total biomass	Grazer	1	421.15	4.293	0.072	0.14
	Temp × grazer	1	4.40	0.045	0.837	
	Error	8	1569.6			
Microalgae	Temp	1	0.002	8.973	0.017	0.35
Growth	Grazer	1	0.001	4.489	0.067	0.15
	Temp × grazer	1	0.0001	0.290	0.605	
	Error	8	0.0002			

**Table 2:** ANOVA results explaining the effects of temperature (temp) on total grazer abundance (ab) and total grazer biomass (bm) (mg AFDW without shell) as well as on the abundance (ab) and the per capita biomass (per capita bm) (mg AFDW individual^−1^ without shell) of each grazer taxon (*Gammarus* spp., *Idotea* spp. and *L. littorea*). Sample size (*N*) was six

Variable	Factor	*df*	MS	*F*	*p*
Total grazer ab	Temp	1	4266.7	15.591	0.017
	Error	4	273.7		
Total grazer bm	Temp	1	914,311	13.706	0.021
	Error	4	66,709		
*Gammarus* spp. ab	Temp	1	5766.0	12.687	0.024
	Error	4	454.5		
*Gammarus* spp. per capita bm	Temp	1	0.085	0.074	0.072
	Error	4	0.014		
*Idotea* spp. ab	Temp	1	13.50	3.857	0.121
	Error	4	3.50		
*Idotea* spp. per capita bm	Temp	1	73.51	15.374	0.017
	Error	4	4.781		
*L. littorea* ab	Temp	1	48.17	2.035	0.227
	Error	4	23.67		
*L. littorea* per capita bm	Temp	1	3260.04	20.876	0.010
	Error	4	156.16		

**Fig. 4:**
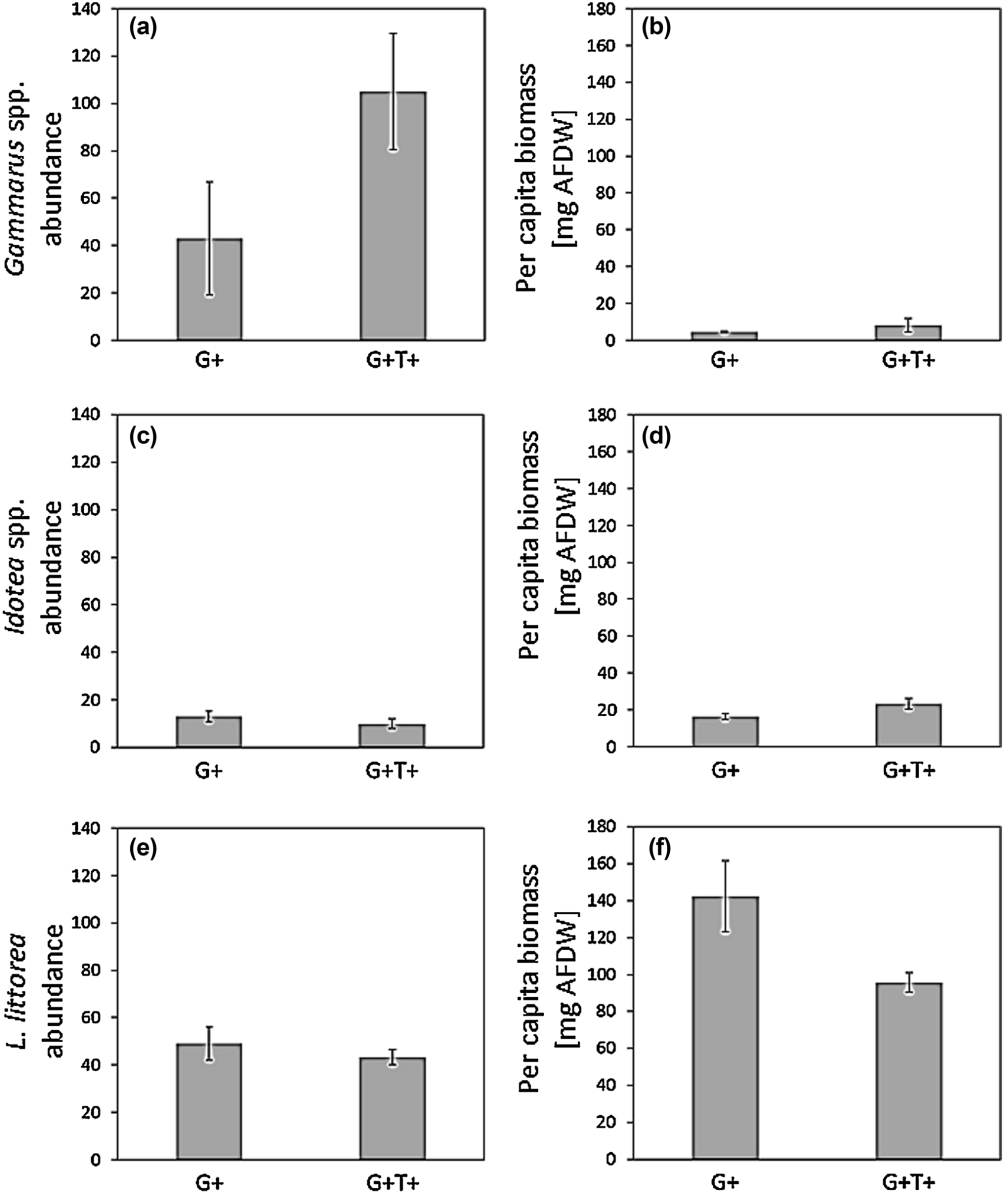
Display (mean ± CI) of the mesograzer taxon-specific abundance and per capita biomass (mg AFDW without shell) for *Gammarus* spp. (**a, b**), *Idotea* spp. (**c, d**) *L. littorea* (**e, f**) in ambient (+G) and high temperature (+G+T) treatments. Sample size (*N*) was six

Analyses of the grazer taxa specific responses to warming showed that the positive main effects on total mesograzer abundance and biomass were driven by positive effects on both crustacean mesograzer taxa. Precisely, warming significantly increased the abundance of *Gammarus* spp. and showed a trend of a positive effect on its per capita biomass (Fig. 4a, b; Table 2). Moreover, warming significantly increased the per capita biomass of *Idotea* spp. (Fig. 4c, d; Table 2). In contrast to this, warming decreased the per capita biomass of the gastropod *L. littorea* (Fig. 4e, f; Table 2).

A priori planned comparison assessing the direct effect of temperature on microalgal total biomass and growth rate in non-grazed or grazed treatments indicated a significant positive effect of warming under grazed conditions (i.e., +G vs. +T+G) prior but not after alpha correction (Table 3). In non-grazed treatments (i.e., −G vs. +T−G) it indicated a trend of a positive effect of warming on microalgal total biomass (Table 3). With 75.0 µg Chl *a* cm^−2^ and 65.4 µg Chl *a* cm^−2^ in grazed and non-grazed treatments, respectively, the absolute gain of algal biomass in response to warming was nearly the same. In relative terms, however, microalgal biomass increased by threefold under warm and grazed conditions (from on average +G = 40.4 µg Chl *a* cm^−2^ to on average +T+G = 115.4 µg Chl *a* cm^−2^, Fig. 2), whereas it only doubled under warm and non-grazed conditions (from on average −G = 92.6 µg Chl *a* cm^−2^ to on average +T−G = 158 µg Chl *a* cm^−2^, Fig. 2).

**Table 3:** A priori planned comparisons explaining the effects of grazers and temperature on microalgal biomass (µg cm^−2^ Chl *a*) and the effects of temperature on microalgal growth (µg cm^−2^ Chl *a* day^−1^). Grazer effects on microalgal biomass were tested by comparing non-grazed with grazed treatments in either ambient (i.e., −G vs. +G) or high temperature (i.e., +T−G vs-+T+G) treatments. Temperature effects on microalgal biomass and growth were tested by comparing ambient and high temperature treatments under either non-grazed (i.e., −G vs. +T−G) or grazed (i.e., +G vs. +T+G) conditions. Sample size (*N*) twelve. Shown are the *p* values of the planned comparison and the significance level required after Bonferroni correction

Variable	Comparison	MS	*F*_1, 8_	*P*	Adjusted significance level
Microalgal	−G vs. +G	255.81	2.608	0.145	0.0125
Total biomass	+T+G vs. +T−G	169.74	1.730	0.225	0.0125
	+G vs. +T+G	527.97	5.382	0.049	0.0125
	−G vs. +T−G	400.47	4.082	0.078	0.0125
Microalgae	+G vs. +T+G	4.27	6.650	0.033	0.025
Growth	−G vs. +T−G	1.67	2.603	0.145	0.025

A priori planned comparison assessing the indirect temperature effect via mesograzers on microalgal total biomass turned out non-significant in either ambient or high temperature treatments (i.e. +G vs. −G and +T+G vs. +T−G) prior and after alpha correction (Table 3). Nevertheless, grazing reduced the biomass of microalgae by on average 52 µg Chl *a* cm^−2^ in ambient, and by on average 43 µg Chl *a* cm^−2^ in warm treatments (Fig. 2).

## Discussion

The results show that in spring direct effects of seawater warming constitute a more important determinant of microalgal total biomass accumulation and growth rate than indirect effects via altered top-down grazing. In fact, they indicate that in spring combined elevated temperature and grazing can benefit microalgal biomass accrual. The outcome of this study adds information to previous findings by suggesting that not only the effect direction and size of seawater warming vary with season (compare Werner et al. 2016a), but also the relative importance of the underlying direct or indirect pathways of the effect.

In the experiment seawater warming had a direct positive effect on microalgal biomass accumulation and growth rate, which was not offset by the presence of mesograzers (partially accepting hypothesis 1). Moreover, seawater warming had a positive effect on total mesograzer abundance and biomass (accepting hypothesis 2). Contrary to expectations, however, the positive effect of warming on mesograzers did not lead to a stronger depletion of algal total biomass (rejecting hypothesis 3). This outcome suggests that the positive effect of temperature on mesograzers did not translate into significantly higher grazing pressure on microalgae or, if it did, that the direct positive effect of warming on microalgal growth and total biomass accrual exceeded the negative (indirect) effect of enhanced top-down forcing. Both could be explained by the observed mesograzer taxon-specific effects of warming (accepting hypothesis 4) and by season.

The applied grazer taxa naturally co-occur in *F. vesiculosus* belts of the southwestern Baltic Sea. They are known to feed on benthic microalgae, filamentous macroalgae, and the foundation seaweed *F. vesiculosus* with, however, differences in their feeding mode and, thus, efficiency (Lotze 1998; Sommer 1999a, b). More precisely, feeding of the crustaceans *Gammarus* spp. and *Idotea* spp. is described as picking and lawn-mowing, which does not fully remove microalgal biofilms from the substrate (Sommer 1999a). In contrast, feeding by the gastropod *L. littorea* is described as bulldozer-like and more efficient in clearing microalgal biofilms (Sommer 1999a, b, 2000). Warming significantly increased the total abundance and biomass of mesograzers, which indicates enhanced feeding, growth and reproduction and conforms to MTE predictions stating accelerated metabolism-associated processes in heterotrophs under warming (e.g., Brown et al. 2004; Allen et al. 2005). The response of grazers to warming, however, varied taxon-specifically which can be attributed to the differences in their life history strategies.

The abundance of *Gammarus* spp. increased by nearly threefold, pointing to enhanced recruitment under warming in spring that matches a nearly all-season reproductive pattern described for the different species of *Gammarus* in the Baltic Sea (Kolding and Fenchel 1979; Welton and Clarke 1980). In contrast to this, the per capita biomass of *Idotea* spp. doubled, indicating higher individual growth instead of recruitment in warm treatments. This response conforms with life cycle characteristics of the isopod that describe a somatic growth phase in spring prior to recruitment in early summer (Salemaa 1979; Kroer 1989). Contrasting the positive effects on both crustacean mesograzers, warming decreased the per capita biomass of *L. littorea*. Considering that the gastropod was found irresponsive to much higher temperatures (Clarke et al. 2000; Werner et al. 2016a, b), however, this effect may be explained by an earlier onset of egg spawning of *L. littorina* under warming rather than by physiological constraints. Spawning of *L. littorea* has generally been described to occur in spring, to be influenced by temperature (Graham 1975), and to be linked to a change in body weight in female specimen (Graham 1973).Overall, these results on grazer taxon-specific responses to warming reveal that an increase in grazing pressure (as suggested by the increase in total grazer abundance and biomass) was driven by the positive effects on both crustacean grazers, of which the grazing impact was possibly not sufficient to counteract the enhanced biomass accrual of microalgae under warming in spring. Instead their feeding modes may have even promoted algal growth by re-opening space without fully clearing the substrate from microalgal cells.

The experiment was conducted in spring, which in temperate regions such as the Kiel Fjord is characterized by blooming of marine autotrophs, because inorganic nutrients, light intensity, photoperiod and temperature constitute less limiting abiotic constraints. At the onset of the experiment, inorganic nutrient concentrations comprised about 15 µmol L^−1^ total dissolved inorganic N (including nitrate, nitrite and ammonia), 0.5 µmol L^−1^ Phosphate and 10 µmol L^−1^ Silicate and day length was about 11 h. The seawater manipulation by delta 5 °C resulted in a relatively constant temperature regime between 10 and 12 °C in warm treatments, which naturally occurs later in spring or early summer at the experimental site (Kiel Fjord monitoring data 2007–2013, Webers et al. unpubl data). The temperature coefficient Q10 for marine microalgae in non-limiting conditions is generally assumed to describe a value near 2, meaning that photosynthesis and the associated cell-division double for each 10 °C increase until unfavorable conditions are reached (Eppley 1972; Admiraal 1976; Raven and Geider 1988). Given that benthic microalgae in coastal habitats of the temperate Baltic Sea are adapted to wide temperature ranges across seasons (>30 °C), it can be assumed that warming by delta 5 °C led to more favorable thermal conditions in spring (rising from 5 to 7 °C to 10–12 °C) and that the microalgal community was able to make rapid use of the available resources via temperature-driven faster growth and higher biomass accumulation which exceeded the counteracting effects of grazing.

Altogether, the simultaneous manipulation of one abiotic (temperature) and one biotic (grazing) factor allowed disentangling the relative importance of the direct effects of both factors on microalgal biomass accrual as well as the indirect effects of abiotic change (temperature) through altered trophic interactions (grazing). The results show that seawater warming in spring has a stronger direct than indirect effect on microalgal biomass accumulation in the *F. vesiculosus* system, which implies that bottom-up instead of top-down processes constitute a more important driver of benthic microalgal biomass during this time of the year. The outcome contrasts findings in other macrophyte systems, where grazing mediated or compensated for the effects of environmental change on benthic microalgae or turf production (Alsterberg et al. 2013; Ghedini et al. 2015). Considering that top-down grazing forms a crucial structuring force in coastal vegetated systems in general, it is possible that these contrasting findings are due to season rather than intersystem differences in compensatory mechanisms. More precisely, mediation of effects by consumers was observed in late summer (Alsterberg et al. 2013), when grazing pressure in temperate systems is generally highest, and in early spring (Ghedini et al. 2015), possibly during pre-bloom conditions for micro- and filamentous algae. Related to this, it can be assumed that in the temperate *F. vesiculosus* system the relative importance of the effective pathways switches (i.e., the direct temperature effect weakens and the indirect, compensatory effect through altered grazing strengthens) during seasonal succession towards summer, as soon as the carrying capacity of the system is reached and other resources (e.g., nutrients, space, light) limit the temperature-accelerated microalgal biomass accrual (e.g., O’Connor et al. 2009). Previous work on the experimental *F. vesiculosus* system showed, however, that in summer the same positive effect of warming on ephemeral algae (here: micro- and filamentous macroepiphytes) can be triggered via indirect pathways (here: loss of top-down grazing) with deleterious effects on the foundation seaweed (Werner et al. 2016a, b). In this context, the study adds mechanistic information to the overarching goal of understanding and predicting the seasonal variability of climate change effects. The outcome of this work and previous studies suggest that the relative importance of the underlying direct and indirect effective pathways of warming and the consequent effect on the ecological balance between production and consumption are interlinked with the relative importance of the regulating top-down and bottom-up forces, which in the temperate region is related to season.

## Electronic supplementary material

Below is the link to the electronic supplementary material.

Supplementary material 1 (PDF 117 KB)

## References

[CR1] Admiraal W (1976). Influence of light and temperature on the growth rate of estuarine benthic diatoms in culture. Mar Biol.

[CR2] Allen AP, Gillooly JF, Brown JH (2005). Linking the global carbon cycle to individual metabolism. Funct Ecol.

[CR3] Alsterberg C, Eklöf JS, Gamfeldt L, Havenhand JN, Sundbäck K (2013). Consumers mediate the effects of experimental ocean acidification and warming on primary producers. P Natl Acad Sci USA.

[CR4] BACC Author Group (2008). Assessment of climate change for the Baltic Sea Basin—Regional Climate Studies.

[CR5] BACC Author Group (2015). Second assessment of climate change for the Baltic Sea Basin.

[CR6] Brown JH, Gillooly JF, Allen AP, Savage VM, West GB (2004). Toward a metabolic theory of ecology. Ecology.

[CR7] Burkepile DE, Hay ME (2006). Herbivore vs. nutrient control of marine primary producers: Context-dependent effects. Ecology.

[CR8] Carr LA, Bruno JF (2013). Warming increases the top-down effects and metabolism of a subtidal herbivore. PeerJ.

[CR9] Clarke AP, Mill PJ, Grahame J (2000). The nature of heat coma in *Littorina littorea* (Mollusca: Gastropoda). Mar Biol.

[CR10] Eppley RW (1972). Temperature and Phytoplankton growth in the sea. Fish B-NOAA.

[CR11] Falkenberg LJ, Connell SD, Russell BD (2014). Herbivory mediates the expansion of an algal habitat under nutrient and CO_2_ enrichment. Mar Ecol Progr Ser.

[CR12] Ghedini G, Russell BD, Connell SD (2015). Trophic compensation reinforces resistance: herbivory absorbs the increasing effects of multiple disturbances. Ecol Lett.

[CR13] Grahame J (1973). Breeding energetics of *Littorina littorea* (L.) (Gastropoda: Prosobranchiata). J Anim Ecol.

[CR14] Grahame J (1975). Spawning in *Littorina littorea* (L.) (Gastropoda: Prosobranchiata). J Exp Mar Biol Ecol.

[CR15] HELCOM (2007) Climate Change in the Baltic Sea Area—HELCOM Thematic Assessment in 2007. Balt Sea Environ Proc

[CR16] Jeffrey SW, Humphrey GF (1975). New Spectrophotometric equations for determining Chlorophylls a, b, c_1_ and c_2_ in higher plants, algae and natural phytoplankton. Biochem Physiol Pfl.

[CR17] Kolding S, Fenchel TM (1979). Coexistence and life cycle characteristics of five species of the amphipod genus *Gammarus*. Oikos.

[CR18] Kordas RL, Harley CDG, O’, Connor MI (2011). Community ecology in a warming world: The influence of temperature on interspecific interactions in marine systems. J Exp Mar Biol Ecol.

[CR19] Kroer N (1989). Life-cycle characteristics and reproductive patterns of *Idotea* spp (Isopoda) in the Limfjord, Denmark. Ophelia.

[CR20] Lebreton B, Richard P, Galois R, Radenac G, Pfleger C, Guillou G, Mornet F, Blanchard GF (2011). Trophic importance of diatoms in an intertidal *Zostera noltii* seagrass bed: Evidence from stable isotope and fatty acid analyses. Estuar Coast Shelf S.

[CR21] Lopez-Urrutia A, San Martin E, Harris RP, Irigoien X (2006). Scaling the metabolic balance of the oceans. P Natl Acad Sci USA.

[CR22] Lotze HK (1998) Populations dynamics and species interactions in macroalgal blooms: abiotic versus biotic control at different life-cycle stages. Dissertation, Institut für Meereskunde and der Christian-Albrecht-Universität, Kiel, Germany

[CR23] Miller DC, Geider RJ, MacIntyre HL (1996). Microphytobenthos: the ecological role of the “Secret Garden” of unvegetated, shallow-water marine habitats. I1. Role in sediment stability and shallow-water food webs. Estuaries.

[CR24] Moran MD (2003). Arguments for rejecting the sequential Bonferroni in ecological studies. Oikos.

[CR25] O’Connor MI (2009). Warming strengthens an herbivore-plant interaction. Ecology.

[CR26] O’Connor MI, Piehler MF, Leech DM, Anton A, Bruno JF (2009). Warming and resource availability shift food web structure and metabolism. PloS Biol.

[CR27] Olejnik S, Algina J (2003). Generalized eta and omega squared statistics: measures of effect size for some common research designs. Psychol Methods.

[CR28] Rall BC, Vucic-Pestic O, Ehnes RB, Emmerson M, Brose U (2010). Temperature, predator–prey interaction strength and population stability. Glob Change Biol.

[CR29] Raven JA, Geider RJ (1988). Temperature and algal growth. New Phytol.

[CR30] Salemaa H (1979). Ecology of *Idotea* spp (Isopoda) in the northern Baltic. Ophelia.

[CR31] Sand-Jensen K (1977). Effect of epiphytes on eelgrass photosynthesis. Aquat Bot.

[CR32] Schernewski G, Hofstede J, Neumann T (2010). Global change and Baltic coastal zones. series: Coastal Research Library.

[CR33] Schiel DR, Steinbeck JR, Foster MS (2004). Ten years of induced ocean warming causes comprehensive changes in marine benthic communities. Ecology.

[CR34] Schramm W, Nienhuis PH, Schramm W, Nienhuis PH (1996). The Baltic Sea and its transitionsl zones. Marine benthic vegetation—recent changes and the effects of eutrophication.

[CR35] Sommer U (1999). The susceptibility of benthic microalgae to periwinkle (*Littorina littorea*, Gastropoda) grazing in laboratory experiments. Aquat Bot.

[CR36] Sommer U (1999). The impact of herbivore type and grazing pressure on benthic microalgal diversity. Ecol Lett.

[CR37] Sommer U (2000). Benthic microalgal diversity enhanced by spatial heterogeneity of grazing. Oecologia.

[CR38] Sommer U, Lengfellner K (2008). Climate change and the timing, magnitude, and composition of the phytoplankton spring bloom. Glob Change Biol.

[CR39] Sommer U, Lewandowska A (2011). Climate change and the phytoplankton spring bloom: warming and overwintering zooplankton have similar effects on phytoplankton. Glob Change Biol.

[CR40] Steneck RS, Watling L (1982). Feeding capabilities and limitation of herbivorous mollusks—a functional-group approach. Mar Biol.

[CR41] Traill LW, Lim MLM, Sodhi NS, Bradshaw CJA (2010). Mechanisms driving change: altered species interactions and ecosystem function through global warming. J Anim Ecol.

[CR42] Underwood GJC, Kromkamp J (1999). Primary production by phytoplankton and microphytobenthos in estuaries. Adv Ecol Res.

[CR43] Valentine J, Duffy JE, Larkum A, Orth RJ, Duarte C (2006). The Central Role of Grazing in Seagrass Ecology. Seagrasses: Biology, Ecology and Conservation.

[CR44] Wahl M, Buchholz B, Winde V, Golomb D, Guy-Haim T, Müller J, Rilov G, Scotti M, Böttcher ME (2015). A mesocosm concept for the simulation of near-natural shallow underwater climates: The Kiel Outdoor Benthocosms (KOB). Limnol Oceanogr-Meth.

[CR45] Wallentinus I (1984). Comparisons of nutrient-uptake rates for Baltic macroalgae with different thallus morphologies. Mar Biol.

[CR46] Welton JS, Clarke RT (1980). Laboratory studies on the reproduction and growth of the amphipod, *Gammarus Pulex* (L.). J Anim Ecol.

[CR47] Werner FJ, Graiff A, Matthiessen B (2016). Temperature effects on seaweed-sustaining top-down control vary with season. Oecologia.

[CR48] Werner FJ, Graiff A, Matthiessen B (2016). Even moderate nutrient enrichment negatively adds up to global climate change effects on a habitat-forming seaweed system. Limnol Oceanogr.

[CR49] Worm B, Lotze HK, Böstrom C, Engkvist V, Labanauskas V, Sommer U (1999). Marine diversity shift linked to interactions among grazers, nutrients and propagule banks. Mar Ecol Prog Ser.

[CR50] Worm B, Lotze HK, Sommer U (2000). Coastal food web structure, carbon storage, and nitrogen retention regulated by consumer pressure and nutrient loading. Limnol Oceanogr.

