# Supplementary material for: Warming has stronger direct than indirect effects on benthic microalgae in a seaweed system in spring
Source: Mar Biol. 2017 Mar 6;164(4):67. doi: 10.1007/s00227-017-3109-x (PMC5337517; doi:10.1007/s00227-017-3109-x)

Marine Biology

Warming has stronger direct than indirect effects on benthic microalgae in a seaweed system in spring

Franziska Julie Werner[1]*, Birte Matthiessen[1]

[1]GEOMAR Helmholtz Centre for Ocean Research Kiel

Experimental Ecology and Food Webs

Düsternbrooker Weg 20, 24105 Kiel, Germany

*Corresponding author: fwerner@geomar.de

phone: +49 431 600 4407

fax +49 431 600 1515

Electronic Supplementary Material 1

In a series of experiments in 2014/15 both *F. vesiculosus* thalli and ceramic tiles were deployed in benthic mesocosms and were sampled in parallel. Shown are the community composition and the mean relative contribution (%) of microalgal species to total biomass on the surface of *F. vesiculosus* and on ceramic tiles in ambient conditions after four weeks of experimental runtime in February 2014. Sample size (N) was six

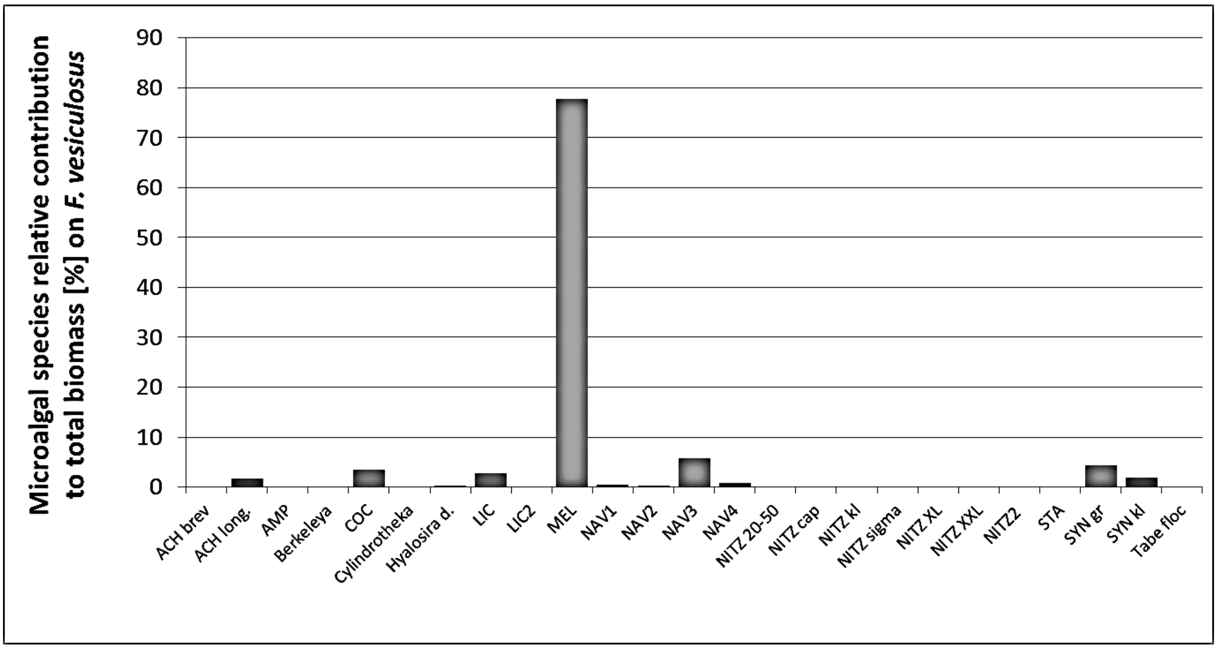

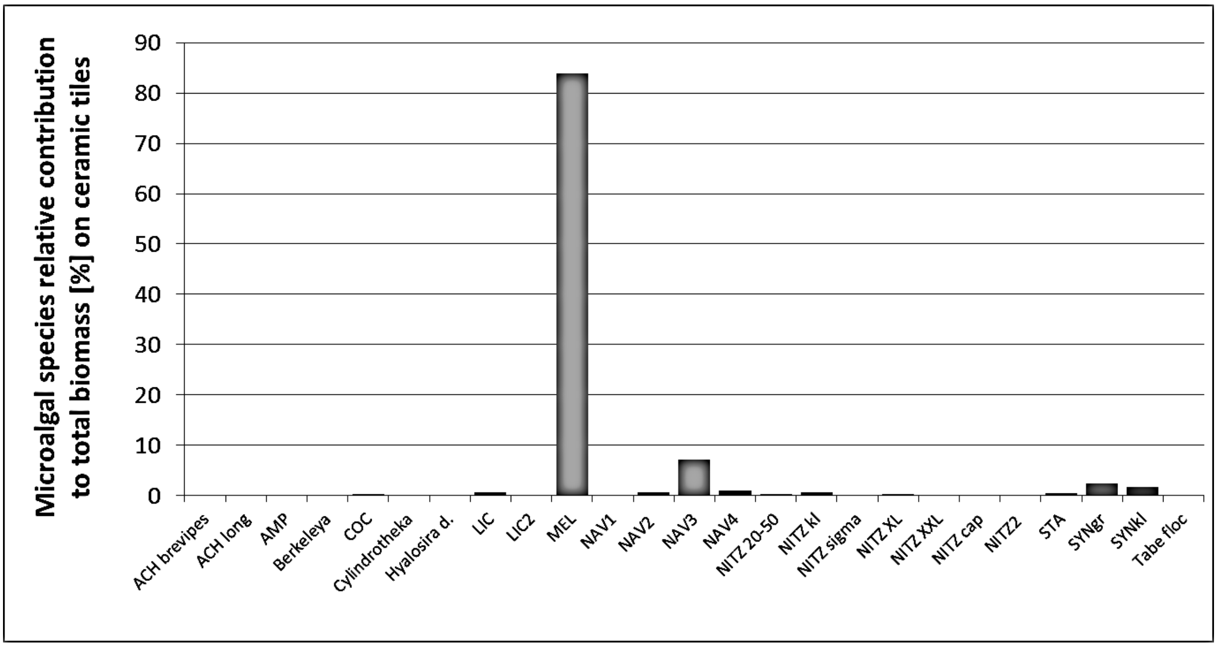

Electronic Supplementary Material 2

Shown are the diversity indices (mean ± CI) of the microalgal community on the surface of ceramic tiles and on *F. vesiculosus*. Sample size (N) was six.

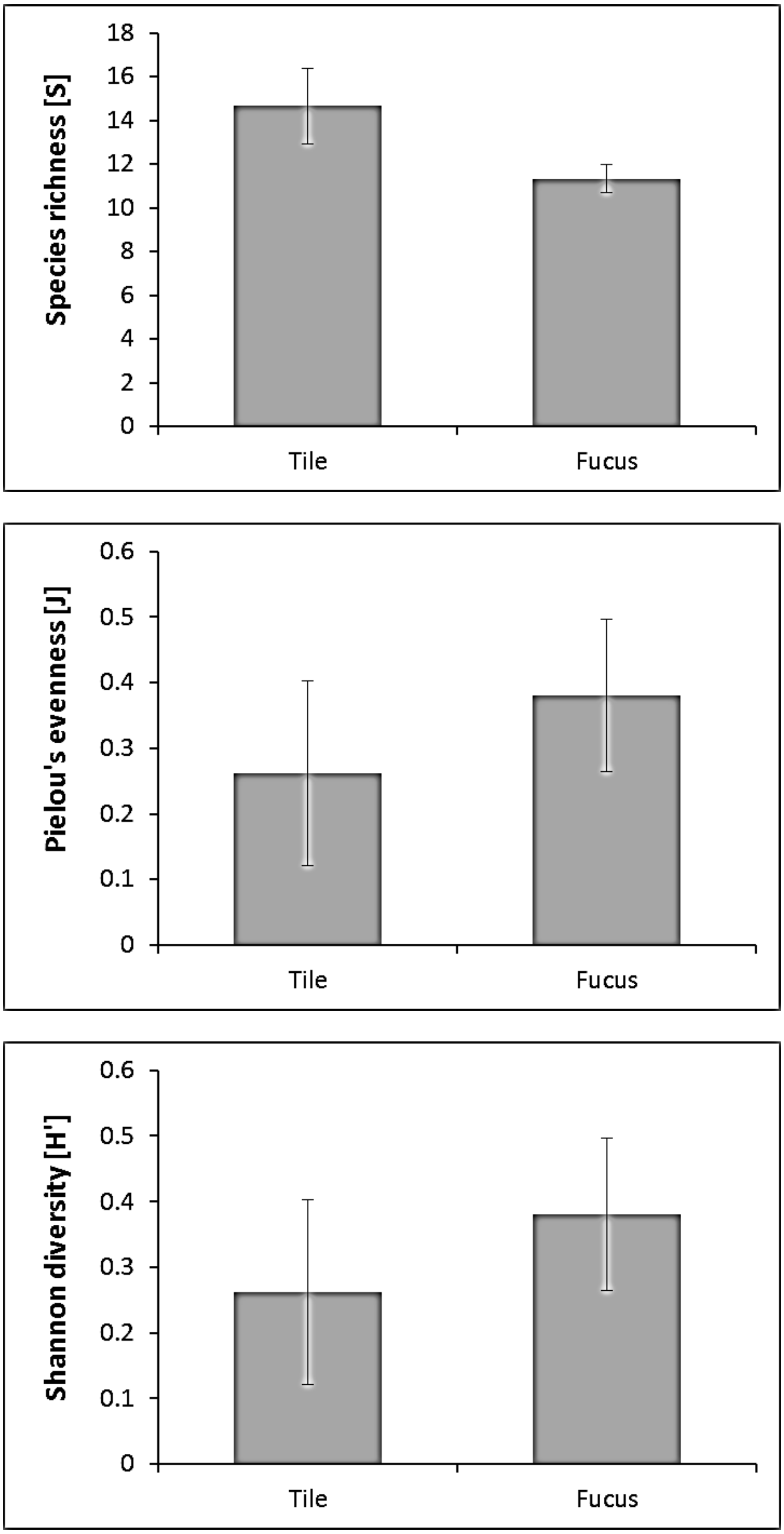

Electronic Supplementary Material 3

Shown is the total biomass accumulation (mean ± CI) of benthic microalgae on the surface of ceramic tiles and *F. vesiculosus* in ambient conditions after four weeks of experimental runtime (February 2014). Biomass is shown as biovolume calculated as pg C per 1 tile or per 1 g DW of *F. vesiculosus*. Sample size (N) was six. It has to be noted that this comparison has to be treated with caution as 1 g DW of *F. vesiculosus* only represents an approximation of the surface area of the ceramic tiles (4.5 x 4.5cm).

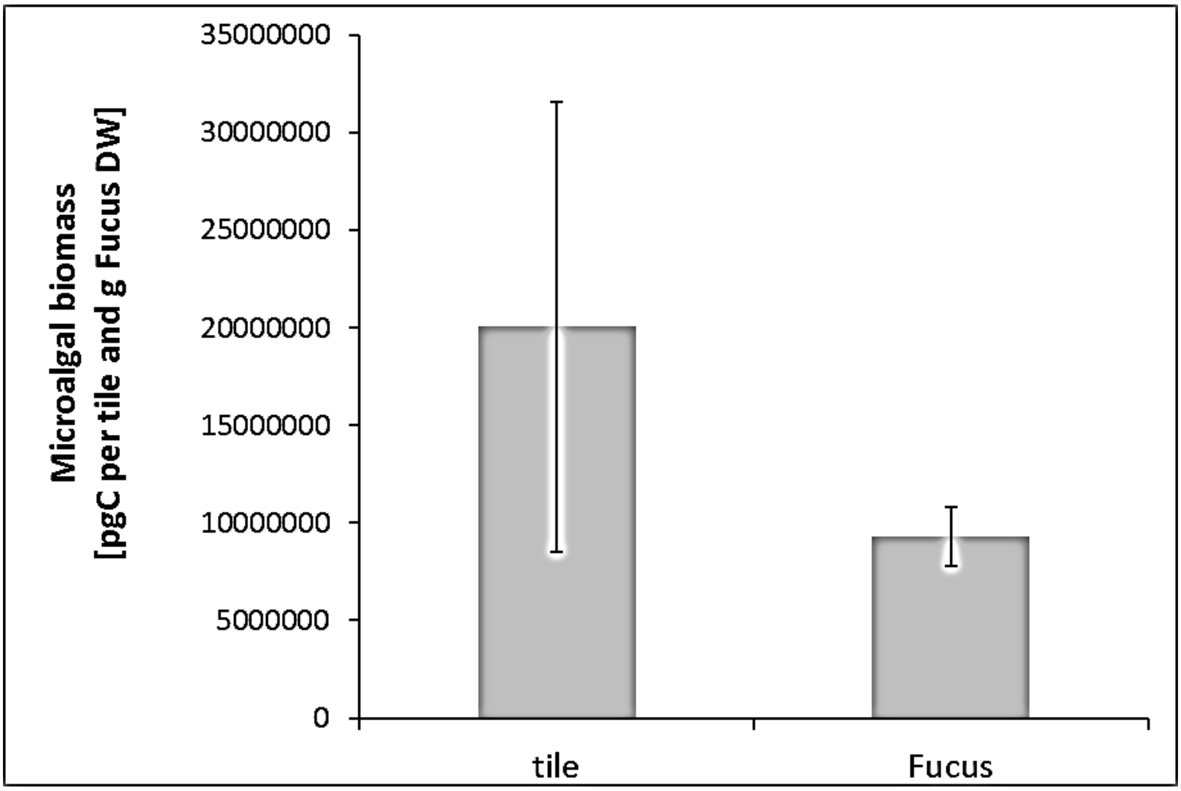

Supplement: Supplementary file 1 — Supplementary material 1 (PDF 117 KB) [file 227_2017_3109_MOESM1_ESM.pdf]
